# Development of temporal modelling for forecasting and prediction of malaria infections using time-series and ARIMAX analyses: A case study in endemic districts of Bhutan

**DOI:** 10.1186/1475-2875-9-251

**Published:** 2010-09-03

**Authors:** Kinley Wangdi, Pratap Singhasivanon, Tassanee Silawan, Saranath Lawpoolsri, Nicholas J White, Jaranit Kaewkungwal

**Affiliations:** 1 Department of Tropical Hygiene, Faculty of Tropical Medicine, Mahidol University, Bangkok, Thailand; 2 Yebilaptsa General Hospital, Zhemgang, Bhutan; 3 The Rural Health Training and Research Centre, Faculty of Public Health, Mahidol University, Bangkok, Thailand; 4 Mahidol-Oxford Unit, Faculty of Tropical Medicine, Mahidol University, Bangkok, Thailand

## Abstract

**Background:**

Malaria still remains a public health problem in some districts of Bhutan despite marked reduction of cases in last few years. To strengthen the country's prevention and control measures, this study was carried out to develop forecasting and prediction models of malaria incidence in the endemic districts of Bhutan using time series and ARIMAX.

**Methods:**

This study was carried out retrospectively using the monthly reported malaria cases from the health centres to Vector-borne Disease Control Programme (VDCP) and the meteorological data from Meteorological Unit, Department of Energy, Ministry of Economic Affairs. Time series analysis was performed on monthly malaria cases, from 1994 to 2008, in seven malaria endemic districts. The time series models derived from a multiplicative seasonal autoregressive integrated moving average (ARIMA) was deployed to identify the best model using data from 1994 to 2006. The best-fit model was selected for each individual district and for the overall endemic area was developed and the monthly cases from January to December 2009 and 2010 were forecasted. In developing the prediction model, the monthly reported malaria cases and the meteorological factors from 1996 to 2008 of the seven districts were analysed. The method of ARIMAX modelling was employed to determine predictors of malaria of the subsequent month.

**Results:**

It was found that the ARIMA (p, d, q) (P, D, Q)^s ^model (p and P representing the auto regressive and seasonal autoregressive; d and D representing the non-seasonal differences and seasonal differencing; and q and Q the moving average parameters and seasonal moving average parameters, respectively and s representing the length of the seasonal period) for the overall endemic districts was (2,1,1)(0,1,1)^12^; the modelling data from each district revealed two most common ARIMA models including (2,1,1)(0,1,1)^12 ^and (1,1,1)(0,1,1)^12^. The forecasted monthly malaria cases from January to December 2009 and 2010 varied from 15 to 82 cases in 2009 and 67 to 149 cases in 2010, where population in 2009 was 285,375 and the expected population of 2010 to be 289,085. The ARIMAX model of monthly cases and climatic factors showed considerable variations among the different districts. In general, the mean maximum temperature lagged at one month was a strong positive predictor of an increased malaria cases for four districts. The monthly number of cases of the previous month was also a significant predictor in one district, whereas no variable could predict malaria cases for two districts.

**Conclusions:**

The ARIMA models of time-series analysis were useful in forecasting the number of cases in the endemic areas of Bhutan. There was no consistency in the predictors of malaria cases when using ARIMAX model with selected lag times and climatic predictors. The ARIMA forecasting models could be employed for planning and managing malaria prevention and control programme in Bhutan.

## Background

There are an estimated 300-500 million clinical cases of malaria reported each year [1-4]. One factor contributing to this problem is the known global climate change; this is considered as a big challenge in fight against the scourge of malaria [5-7]. In Bhutan, despite many years of prevention and control measures, malaria still remains a public health problem in low lying areas of the country adjacent to India. In some districts, the transmission persistently occurs throughout the year. Besides its impact on social, demographic and economic disruptions, malaria is considered a threat as it may introduce malaria into all temperate climates and non-malarious areas across Bhutan.

Historically, the first malaria survey in Bhutan was conducted in 1962 [8]. There was a steady increase in the number of cases rising from 518 in 1965 to reach a peak of 39,852 cases with 62 deaths in 1994, followed by a decline to 392 cases with two deaths in 2008 as a result of intensive prevention and control measures. The highest number of cases was recorded in 1994 with 39,852 cases, and the highest number of deaths was also recorded in 1994 with 62 deaths. The Annual Parasite Incidence (API) per 1,000 population at risk of malaria in Bhutan was 3.98% in the year 2006 [9]. However, the numbers of cases remain persistently high in seven endemic districts of Bhutan with malaria transmission occurring throughout the year. The seven endemic districts of Bhutan are: Chukha, Dagana, Pemagatshel, Samdrup Jongkhar, Samtse, Sarpang and Zhemgang.

Malaria transmission is clearly associated with the rainy season [10-13]. Prevention and control measures have been intensified during the summer months- May to July, since the transmission is increased during these months. The control measures also have changed over the years. Indoor residual spraying (IRS) using dichlorodiphenyltrichloroethane (DDT) was implemented since 1964 with three rounds and later two rounds until 1994. With reports of resistance to DDT in some parts of the world, and global concern over environmental concerns, DDT was replaced by Deltamethrin (synthetic pyrethoid) from 1995. However, DDT is used in the neighbouring state of Assam in India which borders five of the seven malaria endemic districts of Bhutan, since the main vector *Anopheles minimus *is still sensitive to DDT [14]. Insecticide treated-bed nets (ITN) became the main control strategy from 1998 with focal IRS being used during outbreaks and emergencies, and in high *Plasmodium falciparum *transmission areas with API >10% [8]. With the growing evidence of the effectiveness of LLINs in reducing the mortality and morbidity of malaria by preventing man-mosquito contact [15], from 2006 Bhutan distributed over 100,000 long-lasting insecticidal nets (LLIN), supported by grants from Global Fund to Fight AIDS, Tuberculosis and Malaria (GFATM). Early diagnosis and prompt treatment (EDPT) remains a cornerstone of malaria control and is provided by microscopy facilities at all levels of health centres and supplemented by rapid diagnostic kits (RDT). The present treatment regimen of *Plasmodium falciparum *is the combination of artemether and lumefantrine (Coartem^®^) administered over three days (except for pregnant woman). *Plasmodium vivax *is treated with chloroquine (25 mg/kg) for three days and primaquine (0.25 mg/kg) administered over 14-days [16,17].

Although there has been a marked reduction of malaria cases due to the implementation of these integrated control measures by the VDCP for the whole country, the malaria problem is still a major threat to the country and requires a well developed strategic plan and resource preparation for prevention and control, and eventual elimination. Forecasting malaria enables suitable allocation of resources and forestalls outbreaks and epidemics. There has been no such modelling of malaria in Bhutan before. This study aimed to propose a forecasting model based on the time series analysis. In addition, this study also aimed at proposing a prediction model incorporating climatic factors such as temperature, humidity and rainfall; which are important in the development of malaria parasites and vector bionomics [18-21].

## Methods

### Study area

**Figure 1:**
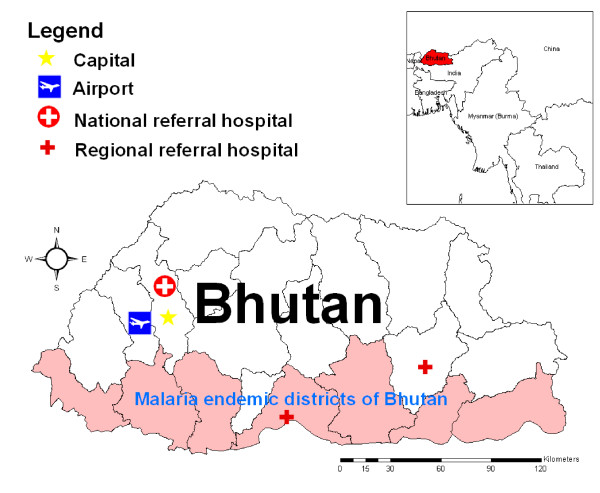
**Study site, map of Bhutan and the endemic districts**.

Seven malaria endemic districts of total 20 districts in Bhutan were selected for this study as they have been identified since malaria transmission occur throughout the year; these districts are Chukha, Dagana, Pemagatshel, Samdrup Jongkhar, Samtse, Sarpang and Zhemgang districts (Figure 1). These districts lie in the foothills of Himalayas neighbouring India. There are four seasons in Bhutan, each season lasts about three months. The general climate in these districts is sub-tropical with heavy rainfall in the summer season lasting from May to July. Malaria transmission is intense during the summer season, the gradual decline in malaria transmission is observed during winter season when the ambient temperature is cooler. The total population of these seven districts, as of 2008, was 277,257. However, populations from different parts of Bhutan usually come to these districts for business and other works because major commercial hubs are located there.

### Malaria data

The monthly incidence of malaria cases were obtained from Vector-borne Disease Control Programme (VDCP), Department of Public Health, Ministry of Health, Royal Government of Bhutan. Malaria cases from various levels of health centres are reported to the programme every month. These health centres provide free malaria diagnosis either by microscopy or by using rapid diagnostic test (RDT). All malaria cases are treated with the national standard regimen.

**Figure 2:**
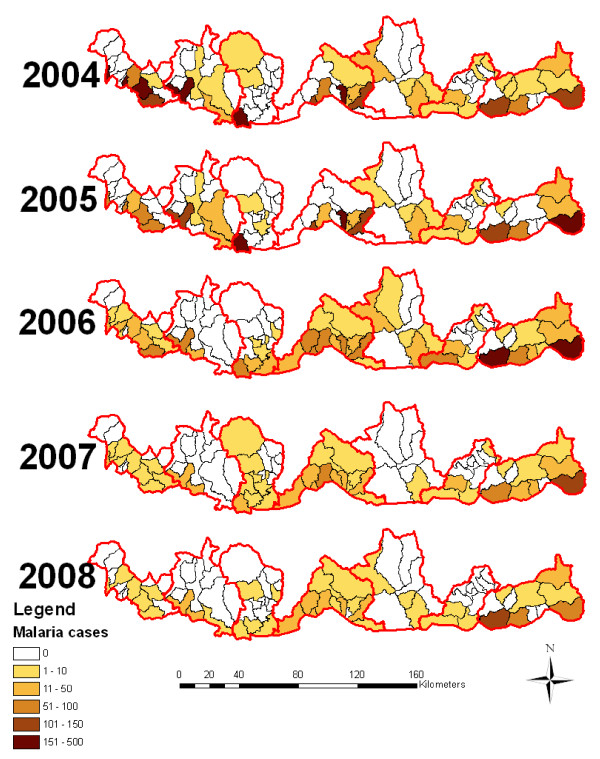
**Malaria case distributions of the endemic sub districts of Bhutan from 2004 to 2008**.

**Figure 3:**
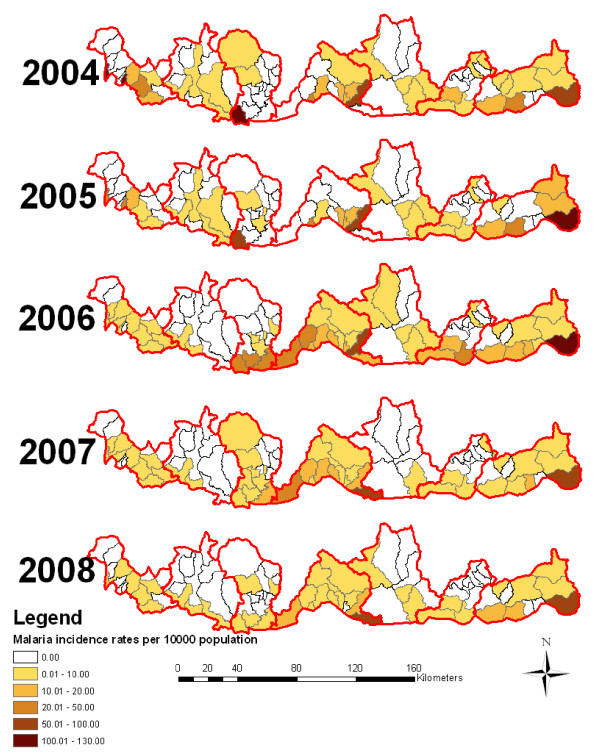
**Malaria incidence rates of the endemic sub districts of Bhutan from 2004 to 2008**.

Malaria distribution varies greatly between the districts and sub districts. Figure 2 and 3 shows the malaria case and incidence rate distribution down to the sub district level with some sub districts reporting as many as 438 cases, where as other some sub districts do not report any malaria cases. Figure 3 shows the malaria incidence rate per 1000 population at the sub district level.

### Meteorological data

**Figure 4:**
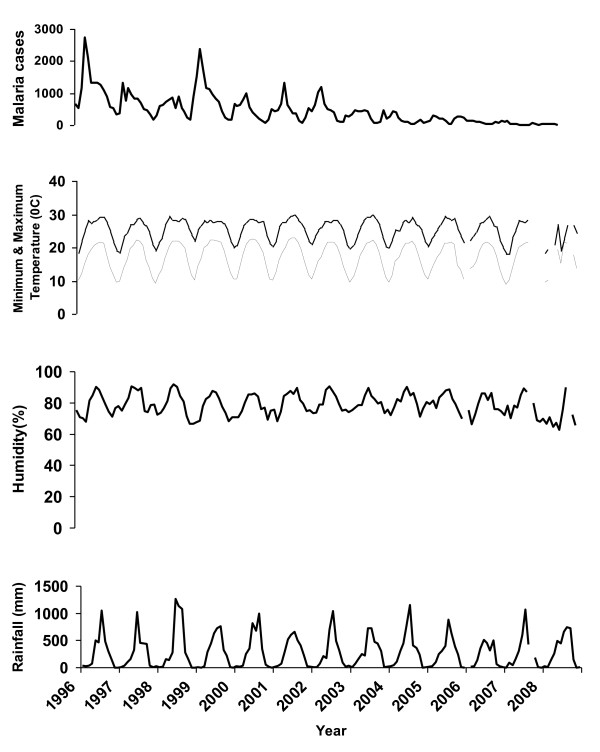
**Monthly malaria cases of the overall districts with temperature, humidity and rainfall from 1996 to 2008**.

A climatic record from 1996 to 2008 was obtained from the Meteorological Unit, Department of Energy, Ministry of Economic Affairs, Royal Government of Bhutan. Daily reported climatic variables include mean minimum and maximum temperature, humidity and rainfall; these variables are collected and recorded at the weather stations in each districts. The meteorological Unit maintains the records of all the district climatic variables at the central level. Overall, the maximum temperature of the whole region varies between 20°C and 30°C; mean minimum temperature ranges between 10°C and 20°C. Monthly relative humidity ranges between 60% and 90%. Amount of rainfall greatly varies from month to month, ranging from zero to 1000 mm per month (Figure 4).

### ARIMA Modelling Methods

The forecasting model proposed in this study was the multiplicative seasonal Auto-regressive Integrated Moving Average (ARIMA). A seasonal ARIMA (p, d, q) (P, D, Q) ^s ^model where:

▪ **p **and **P**- the auto regressive and seasonal autoregressive, respectively;

▪ **d **and **D**- the non-seasonal differences and seasonal differencing, respectively;

▪ **q **and **Q**- the moving average parameters and seasonal moving average parameters, respectively.

▪ **s **representing the length of the seasonal period.

A stationary time series is a time series whose statistics do not change over time. Such statistics are typically the mean and the variance. There are three types of non-stationarity; series that have a non-stationary mean; series that have a non-stationary variance; and series with a periodic or seasonal component [22].

**Figure 5:**
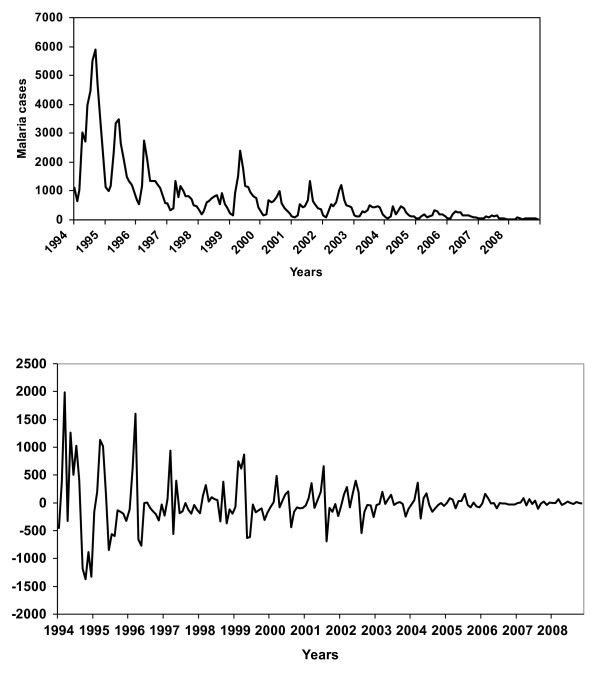
**Malaria cases without any differencing (Top) and with first degree and seasonal differencing of the overall endemic districts of Bhutan from 1994 to 2008**.

In this research, the data series was non-stationary (Figure 5-Top) but after taking first difference, the mean is constant (Figure 5-Bottom). But, there is no evident that the original data series has a variance that gets larger over time. However, in our study non-stationary mean was dealt by taking first differences and a strong seasonal component with a season of length by taking a seasonal difference.

**Figure 6:**
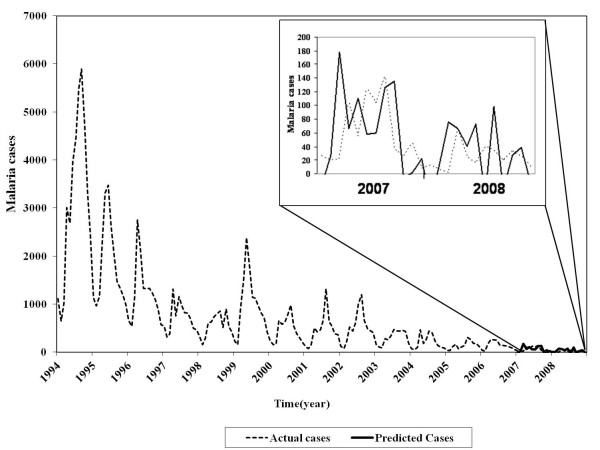
**Actual malaria cases from 1994 to 2008 and predicted cases from 2007 to 2008 of the overall endemic districts of Bhutan**.

From the autocorrelation functions (ACF) and partial autocorrelation functions (PACF), plausible models were identified. The forecasting models were developed for each selected district as well as the overall endemic districts. Time series data from 1994 to 2006 were used as a training set while data from 2007 to 2008 were used as a testing set (Figure 6). The model diagnostic was performed using Bayesian Information Criteria (BIC) and p-value. The lowest BIC values with p value less than 0.05 was considered good model [23-25].

The good ARIMA models from different data series were explored in which the actual cases and predicted cases were closely matched as shown in Figure 6- Bottom. The mean average percentage errors (MAPE) were computed. The best model with the least MAPE was used to forecast the malaria cases for the year 2009 and 2010, respectively.

### ARIMAX Modelling Methods

ARIMAX model is an extension of ARIMA modelling in attempt to predict the malaria cases using the climatic factors and the number of cases in a previous month. The predictors in the model included the number of cases in the previous month, mean maximum and minimum temperature, relative humidity and rainfall lagged at one month.

The modelling of ARIMA and ARIMAX models were performed using STATA Intercooled 9. The use of data was permitted by the Ministry of Health and Ministry of Economic Affairs in Bhutan.

## Results

### Overall malaria incidence

The number of malaria cases of the seven malaria endemic districts decreased from 38,735 in 1994 to 6,781 in 1998. An outbreak of malaria was observed in 1999 when the incidence dramatically increased to 12,109. A significant decrease in malaria cases was observed again since the year 2005. The number of malaria cases fell to a low of 292 in 2008. Malaria distribution also varies greatly across the districts and sub districts (Figure 3). Some sub district reported malaria as many as 438 malaria cases, while some districts did not observe any malaria cases.

### Time-series Forecasting Models

The best-fit models for the different districts and the predicted cases with the actual cases for the year 2007 and 2008 are shown in Table 1. The best-fit model for Chukha and Samdrup Jongkhar districts were ARIMA (2, 1, 1) (0, 1, 1)^12^, where the error percentage were 3.64% and 2.52%, respectively. Dagana, Pemagatshel, Sarpang and Zhemgang district had the best-fit model of ARIMA (1, 1, 1) (0, 1, 1)^12 ^with error percentages of 2.43%, -7.72%, 0.70% and 5.90%, respectively. Samtse had ARIMA (1, 1, 0) (0, 1, 1)^12 ^as the best-fit model with an error percentage of 24.80%. The best-fit model for the overall districts was ARIMA (2, 1, 1) (0, 1, 1)^12^. This had an error percentage of -8.12% (Table 1).

**Table 1:** Actual and predicted malaria cases in endemic districts of Bhutan for two years from 2007 to 2008.

Districts	Model	Malaria cases		Error percentage
			
		Actual cases	Predicted case	
Chukha	(2,1,1)(0,1,1)^12^	80	77.09	3.64
Dagana	(1,1,1)(0,1,1)^12^	51	49.76	2.43
Pemagatshel	(1,1,1)(0,1,1)^12^	41	44.17	-7.72
Samdrup Jongkhar	(2,1,1)(0,1,1)^12^	366	356.78	2.52
Samtse	(1,1,0)(0,1,1)^12^	76	57.16	24.80
Sarpang	(1,1,1)(0,1,1)^12^	386	383.30	0.70
Zhemgang	(1,1,1)(0,1,1)^12^	7	6.59	5.90
All districts	(2,1,1)(0,1,1)^12^	1007.00	1088.80	-8.12

**Figure 7:**
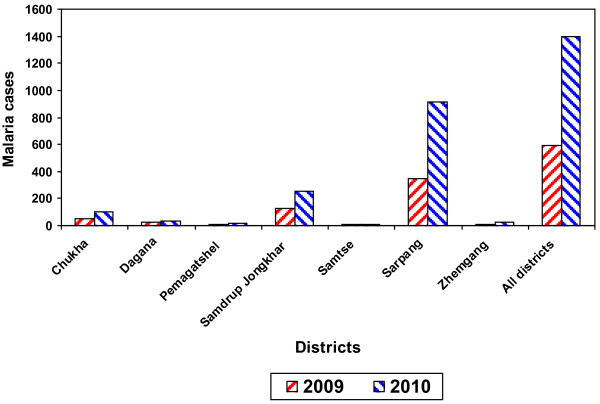
**Forecasted malaria cases of the endemic districts and the overall endemic districts for 2009 and 2010**.

The best model was fitted to forecast the malaria cases for 2009 and 2010. Sarpang district was forecasted to report the highest number of cases with 350 and 915, followed by Samdrup Jongkhar district with 131 and 258 cases, respectively. The lowest cases were forecasted to be reported by Zhemgang district with nine cases for 2009 followed by Samtse district with 11 cases, respectively. In 2010, Samtse was predicted to report the least cases with five followed by Pemagatshel district with 13 cases, respectively (Figure 7 and Tables 2 and 3).

**Table 2:** Forecasted monthly malaria cases of endemic districts from January to December 2009.

District	Forecasted malaria cases												Total
		
	Jan	Feb	Mar	Apr	May	Jun	Jul	Aug	Sep	Oct	Nov	Dec	
Chukha	2	1	2	11	4	5	7	3	3	5	4	4	50
Dagana	1	1	1	4	2	1	2	1	4	3	1	3	24
Pemagatshel	0	1	0	5	0	2	0	1	1	2	0	0	12
Samdrup Jongkhar	3	2	3	18	11	7	19	15	7	21	13	12	131
Samtse	0	0	0	3	2	1	3	2	0	0	0	0	11
Sarpang	14	11	9	39	25	22	33	42	36	38	47	34	350
Zhemgang	0	0	0	1	0	0	2	0	2	1	1	1	9

All district	19	16	15	82	45	40	68	65	53	70	67	55	595

**Table 3:** Forecasted monthly malaria cases of endemic districts from January to December 2010.

District	Forecasted malaria cases												Total
		
	Jan	Feb	Mar	Apr	May	Jun	Jul	Aug	Sep	Oct	Nov	Dec	
Chukha	5	5	5	14	8	9	11	8	7	10	9	9	101
Dagana	2	2	2	5	3	2	3	2	5	4	2	4	35
Pemagatshel	0	1	0	5	0	2	0	1	1	2	0	0	13
Samdrup Jongkhar	10	10	11	27	21	18	30	26	19	33	27	26	258
Samtse	0	0	0	2	1	0	2	1	0	0	0	0	5
Sarpang	48	47	48	80	69	68	81	92	89	94	105	95	915
Zhemgang	1	1	1	2	1	1	4	2	3	2	2	2	22

All district	67	67	70	140	106	104	136	137	128	149	149	140	1393

### Prediction Models with Covariates

The covariates fitted in the models included the number of malaria cases in the previous month, mean maximum and minimum temperature, humidity and rainfall, all lagged at one month period. Pemagatshel and Zhemgang did not have any significant predictors (climatic variables and the previous month's malaria cases) of malaria for the subsequent month. The best model for Chhukha district was the model II with a BIC of 1107.768. The significant predictors was mean minimum temperature (p = 0.002) when the malaria cases of the previous month, mean minimum temperature and humidity lagged at one month were fitted. Previous month's malaria cases were a significant predictor of subsequent month's malaria cases for Dagana districts in all the five models. However, model III was the best model with the lowest BIC of 748.5172. The different predictors that were fitted in the model III were malaria cases, maximum temperature and rainfall. Model III with previous month's malaria cases, maximum temperature and rainfall was the best model for Samdrup Jongkhar district. But two of the covariates, maximum temperature (p < 0.001) and rainfall (p < 0.001) were significant predictors for Samdrup Jongkhar in the Model IV. Samtse, Sarpang and all endemic districts in the model I consisting of previous month's malaria cases, and mean minimum temperature was the best model. However, only significant predictor was mean minimum temperature (Table 4).

**Table 4:** P-values and BIC of significant covariates of malaria case in different ARIMAX models for each of the endemic districts and the overall endemic districts of Bhutan.

Models	Covariates	Districts*					
		
		1	2	4	5	6	All districts
**I**	**Cases**		0.000	0.000	0.000	0.04	0.000
**BIC**	**Temp min**		**792.3911**	**1524.363**	**1337.595**	**1731.596**	**1812.401**

**II**	**Cases,**	0.002	0.000	0.000	0.000	0.033	0.000
**BIC**	**Temp min, Hum**	**1107.768**	**792.5185**	**1528.834**	**1341.371**	**1735.813**	**1815.411**

**III**	**Cases,**	0.001	0.000	0.000 0.000	0.001 0.004	0.038	0.000
**BIC**	**Temp max, Rainfall**	**1112.112**	**784.5172**	**1505.547**	**1343.952**	**1734.896**	**1812.601**

**IV**	**Cases,**		0.000	0.001			
	**Temp max, Rainfall**	0.004		0.000	0.038	0.042	0.000
	**Hum**						
**BIC**		**1126.668**	**796.214**	**1514.975**	**1345.209**	**1744.511**	**1817.437**

**V**	**Cases,**		0.000				
	**Temp max,**			0.001			
	**Temp min, Rainfall,**			0.001			
	**Hum**						
**BIC**			**789.4534**	**1514.97**			

* Districts: 1-Chukha, 2-Dagana, 3-Pemagatshel, 4-Samdrup Jongkhar, 5-Samtse, 6-Sarpang, 7-Zhemgang.

Note:- p-value significant at 0.05;

The blank space means it is not significant at p = 0.05;

Districts 3 and 7 did not have any climatic variables significant at p = 0.05, and thus were not included in the table

Cases-previous month malaria cases, Temp max-monthly mean maximum temperature, Temp min-monthly mean minimum temperature, Hum-humidity;

## Discussion

### Models based on the time series (ARIMA)

This study provides an example of applying a simple ARIMA model to forecast malaria cases in a low malaria-transmission area, where targeted interventions are highly recommended for most effective malaria control. This model was developed according to the trend of the malaria cases over the years and presuming pattern stability of all other conditions such as climatic factors, control and preventive measures, treatment seeking behaviour and migration of people. The developed models were validated and appeared to fit well at both each district level and the overall districts, providing tolerable error levels in forecasting.

Different ARIMA models were found for different districts, which is consistent with the findings of the study by Briet et al [26]. This suggests that each district would have its own particular trend. Districts close to each other are likely to have similar disease transmission patterns corresponding to their spatial and climatic similarity. However, the ARIMA models of the selected endemic districts in Bhutan did not follow this hypothesis in terms of spatial location. In some areas, the high malaria incidence district is adjacent to the very low malaria incidence district. On the other hand, different malaria trends were observed among districts that have similar climatic characteristics. The difference in malaria trends across districts may be partly explained by the difference in control measures or patients' seeking behaviours. The control measures in these endemic districts remain same. However, the actual implementation of the control measures such as IRS may vary from districts to districts. This difference could be due to logistic reasons such as the availability of the people to carry out this activity.

### Models based on time series with covariates (ARIMAX)

In exploring different prediction models by fitting covariates to the time series data, no single best model was found; some covariates were found significant in one model but not in another model. These covariates included number of cases in previous month, mean maximum and minimum temperature, rainfall and humidity. These climatic covariates were lagged at one month to allow for sufficient time to complete life cycle of vector which takes around two weeks and subsequently complete the generation of parasites in the new host for two weeks. The number of malaria cases observed in the previous month reflect the level of human reservoir within the area, while other meteorological factors such as temperature, humidity and rainfall are an important extrinsic factors that are directly associated with the development of vectors. The temperature is also an important factor that determines the rate of development of parasites in the mosquitoes.

Two districts did not have any significant covariates as predictors of malaria cases. This can be explained by the fact that the malaria cases in these districts were rather low with no malaria cases in certain months, therefore the malaria cases of the preceding month was not a significant predictor, or at worst could lead to inaccurate prediction, similar to a study in Sri Lanka [26]. Similar findings in which monthly malaria cases in the previous month were not effective in forecasting were reported by Hay et al [27]. The climatic factors that were not significant predictors particularly in these two districts could be explained such that the meteorological stations collecting climatic factors are from a different location from where malaria cases usually occurred. This indicates as one of the main limitations of using the climatic factors as the predictors for forecasting in certain situation/location [28].

Temperature was found as an important predictor for three districts and overall districts. Temperature affects - the mosquito bionomics through the time required for development of the ookinete, the egg of the parasite, in the midgut of the anopheline mosquito, which decreases as temperature increases from 21°C to 27°C [29]. Increase in temperature also decreases the interval between mosquitoes' blood meals there by shortening the incubation periods of the plasmodium parasites in the mosquitoes and the number of times eggs are laid by the mosquitoes [5,21]. A decrease in temperature would have the opposite effect.

When more climatic variables were added, rainfall was found as one of the significant predictors for only two districts. Rainfall provides aquatic medium for the growth and development of mosquitoes. But excessive rainfall might have a negative effect by washing off the breeding sites [30]. In all, using climatic factors as predictors for malaria occurrence were different from one location to another; this pattern has been observed by several other studies [27,28,30-35].

## Conclusions

These forecasting models developed in the study provide the VDCP with expected malaria cases in advance, which would be a useful guidance for timely prevention and control measures to be effectively planned. The knowledge of the spatial distribution of the malaria at the sub districts would also greatly aid in the targeting the control measures, even though the forecasting is feasible at the district level data due to the small number of cases at the sub district level. Thus, VDCP can use the forecasting model to estimate the number of malaria cases at the district while targeting the control measures at the sub district using the spatial distribution of malaria cases of the previous years.

The prediction model based on the time series and climatic factors were developed and showed different predictors for different districts. Some districts did not have any covariates as predictor of malaria. Model based on time series would provide forecasting for longer period unlike the models fitted with other covariates like climatic factors, which is best suited for forecasting in shorter period.

The main limitation of models based on time series is that they provide forecasting for longer period. In contrast, the models that are fitted with other covariates such as climatic factors may be best suited for forecasting within a shorter period. The time series model proposed in the study can be applied to other diseases such as dengue. However, further research is recommended to evaluate the effectiveness of integrating the forecasting model into the existing malaria control programme in terms of its impact in reducing the disease occurrence and also the cost of control interventions.

## Competing interests

The authors declare that they have no competing interests.

## Authors' contributions

KW and JK conceived the study. KW undertook the statistical analysis, interpretation of results and drafted the manuscript. JW assisted in statistical analysis, interpretation of results and was involved in the critical revision of the manuscript. PS, TS, and SL assisted in statistical analysis, interpretation and revision of the manuscripts. NJW assisted in the interpretation of results and revision of the manuscripts. All authors read and approved the final manuscript.

## References

[B1] Breman JG The ears of the hippopotamus: manifestations, determinants, and estimates of the malaria burden Am J Trop Med Hyg 2001 64 1-2 Suppl 1 11 11425172 10.4269/ajtmh.2001.64.1

[B2] Greenwood BM Bojang K Whitty CJ Targett GA Malaria Lancet 2005 365 1487 1498 10.1016/S0140-6736(05)66420-3 15850634

[B3] Mishra SK Mohapatra S Mohanty S Jaundice in Falciparum Malaria JIACM 2003 4 12 13

[B4] Snow RW Guerra CA Noor AM Myint HY Hay SI The global distribution of clinical episodes of *Plasmodium falciparum *malaria Nature 2005 434 214 217 10.1038/nature03342 15759000 PMC3128492

[B5] Martens WJM Niessen LW Rotmans J Jetten TH McMichael AJ Potential impact of global climate change on malaria risk Environ Health Perspect 1995 103 458 464 10.2307/3432584 7656875 PMC1523278

[B6] Trenberth KE Climate variability and global warming Science 2001 293 48 49 10.1126/science.293.5527.48 11444289

[B7] McMichael AJ Woodruff RE Hales S Climate change and human health: present and future risks Lancet 2006 367 859 869 10.1016/S0140-6736(06)68079-3 16530580

[B8] Vector-borne Disease Control Program Annual report 2007

[B9] WHO Malaria Disease Burden in SEA Region http://www.searo.who.int/EN/Section10/Section21/Section340_4018.htm

[B10] Chaikaew N Tripathi NK Hara S Exploring spatial and spatio-temporal clusters of malaria in Chiang Mai, Thailand International Symposium on Geoinformatics for Spatial Infrastructure Development in earth and Allied Sciences 2008

[B11] Childs DZ Cattadori IM Suwonkerd W Prajakwong S Boots M Spatiotemporal patterns of malaria incidence in northern Thailand Trans R Soc Trop Med Hyg J 2006 100 623 631 10.1016/j.trstmh.2005.09.011 16406037

[B12] Yeshiwondim A Gopal S Hailemariam A Dengela D Patel H Spatial analysis of malaria incidence at the village level in areas with unstable transmission in Ethiopia International Journal of Health Geographics 2009 8 5 10.1186/1476-072X-8-5 19171051 PMC2646707

[B13] Zhou G Sirichaisinthop J Sattabongkot J Jones J Bjornstad ON Yan G Cui L Spatio-temporal distribution of *Plasmodium falciparum *and *P. Vivax *malaria in Thailand Am J Trop Med Hyg 2005 72 256 262 15772317

[B14] Dev V Phookan S Sharma VP Dash AP Anand SP Malaria parasite burden and treatment seeking behavior in ethnic communities of Assam, Northeastern India Journal of Infection 2006 52 131 139 10.1016/j.jinf.2005.02.033 16442438

[B15] Lengerler C Insecticide treated bed nets and curtains for preventing malaria Cochrane review 2004 10.1002/14651858.CD000363.pub2 15106149

[B16] Vector-borne Disease Control Programme Guideline on the management of malaria & vector control in Bhutan, 2007

[B17] Vector-borne Disease Control Programme Bhutan Indicator Framework 2008-2013

[B18] Kirby MJ Lindsay SW Effect of temperature and inter-specific competition on the development and survival of *Anopheles gambiae *sensu stricto and *An. arabiensis *larvae Acta Tropica 2009 109 118 123 10.1016/j.actatropica.2008.09.025 19013420

[B19] Ye Y Louis V Simboro S Sauerborn R Effect of meteorological factors on clinical malaria risk among children: an assessment using village-based meteorological stations and community-based parasitological survey BMC Public Health 2007 7 101 10.1186/1471-2458-7-101 17559638 PMC1913509

[B20] Mabaso MLH Craig M Ross A Smith T Environmental predictors of the seasonality of malaria transmission in Africa: the challenge Am J Trop Med Hyg 2007 76 33 38 17255225

[B21] Bi P Tong S Donald K Parton KA Ni J Climatic variables and transmission of malaria: a 12-year data analysis in Shuchen County, China Public Health Rep 2003 118 65 71 10.1016/S0033-3549(04)50218-2 12604766 PMC1497511

[B22] Dunn P Study Book 2005 The University of Southern Queensland, Australia

[B23] Chafield C The analysis of Time Series: Theory and Practice 1975 Chapman and Hall

[B24] Box GEP Jenkins GW Reinsel GC Time series analysis 1994 Third

[B25] Bowerman B O'Connell R Forecasting and Time Series: An Applied Approach 1987 Third3032450

[B26] Briet OJT Vounatsou P Gunawardena DM Galappaththy GNL Amerasinghe PH Models for short term malaria prediction in Sri Lanka Malaria J 2008 7 76 10.1186/1475-2875-7-76 PMC2412896 18460204

[B27] Hay SI Were EC Reneshaw M Noor AM Ochaola SA Olusanmi L Alipui N Snow RW Forecasting, warning, and detection of malaria epidemics: a case study Lancet 2003 361 1705 1706 10.1016/S0140-6736(03)13366-1 12767739 PMC3164796

[B28] Haghdoost A Alexander N Cox J Modelling of malaria temporal variations in Iran Tropical Medicine & International Health 2008 13 1501 1508 10.1111/j.1365-3156.2008.02166.x 19000157

[B29] Patz JA Strzepek K Lele S Hedden M Greene S Noden B Predicting key malaria transmission factors, biting and entomological inoculation rates, using modeled soil moisture in Kenya Tropical Medicine and International Health 1998 3 818 827 10.1046/j.1365-3156.1998.00309.x 9809915

[B30] Himeidan YE Hamid EE Thalib L Elbashir MI Adam I Climatic variables and transmission of falciparum malaria in New Halfa, eastern Sudan Eastern Mediterramean Health Journal 2007 13 17 24 17546901

[B31] Devi NP Jauhari RK Climatic variables and malaria incidence in Dehradun, Uttaranchal, India J Vect Borne Dis 2006 21 28 16642782

[B32] Gomez-Elipe A Otero A Herp MV Aguirre-Jaime A Forecasting malaria incidence based on monthly case reports and environmental factors in Karuzi, Burundi, 1997-2003 Malaria J 2007 6 129 10.1186/1475-2875-6-129 PMC2048513 17892540

[B33] Mabaso M Vounatsou P Midzi S Da Silva J Smith T Spatio-temporal analysis of the role of climate in inter-annual variation of malaria incidence in Zimbabwe International Journal of Health Geographics 2006 5 20 10.1186/1476-072X-5-20 16700905 PMC1513195

[B34] Briet OJT Vounatsou P Gunawardena DM Galappaththy GNL Amerasinghe PH Temporal correlation between malaria and rainfall in Sri Lanka Malaria J 2008 7 77 10.1186/1475-2875-7-77 PMC2430578 18460205

[B35] Tian L Bi Y Ho S Liu W Liang S Goggins W One-year delayed effect of fog on malaria transmission: a time-series analysis in the rain forest area of Mengla County, south-west China Malaria J 2008 7 11 10.1186/1475-2875-7-110 PMC2441628 18565224

